# Direct detection of polioviruses using a recombinant poliovirus receptor

**DOI:** 10.1371/journal.pone.0259099

**Published:** 2021-11-02

**Authors:** Nancy Gerloff, Mark Mandelbaum, Hong Pang, Nikail Collins, Brittani Brown, Hong Sun, Chelsea Harrington, Jessica Hecker, Chadi Agha, Cara C. Burns, Everardo Vega

**Affiliations:** 1 Division of Viral Diseases, National Center for Immunization and Respiratory Diseases, Centers for Disease Control and Prevention, Atlanta, Georgia, United States of America; 2 Cherokee Nation Assurance, Contracting Agency to the Division of Viral Diseases, Atlanta, Georgia, United States of America; 3 IHRC, Contracting agency to the Division of Viral Diseases, Atlanta, Georgia, United States of America; Institut Pasteur, FRANCE

## Abstract

Polioviruses are positive-sense, single-stranded RNA picornaviruses and the principal cause of poliomyelitis. Global poliovirus surveillance has relied on poliovirus isolation in cells, which may take a minimum of 10 days, involves maintaining two cell lines, and propagates virus in high titers. With eradication underway, a major objective of the Global Polio Eradication Initiative (GPEI) is to develop culture-independent detection of polioviruses as an alternative method to complement the current virus isolation technique. A culture-independent method on poliovirus-positive stool suspensions was assessed with commercially available recombinant soluble poliovirus receptor (PVR) coupled to Histidine (His) tags. Viral RNA was screened by quantitative real-time reverse transcription PCR using the poliovirus intratypic differentiation kit. Poliovirus recovery was optimized with PVR-His–tagged protein and buffers supplemented with polyethylene glycol. To validate the poliovirus-PVR–His tag purification assay, 182 poliovirus-positive stools of programmatic importance were parallel tested against the GPLN-accepted virus isolation method. The PVR-His tag enrichment method detected poliovirus in 164 of 171 poliovirus-positive stools, whereas the virus isolation method misidentified 38 stools as poliovirus-negative (McNemar χ^2^ *p*<0.0001). Using this method in combination with RNA extraction, viral RNA recovery increased and showed similar (WPV1) or higher (Sabin 1) sensitivity than the World Health Organization accredited variation of the virus isolation method. The PVR-His enrichment method could be a viable addition to poliovirus surveillance; similar methods have the potential to capture other human pathogens such as EV71 using an appropriate soluble His tag receptor.

## Introduction

Polioviruses (PV) are positive-sense, single-stranded RNA viruses in the family *Picornaviridae*, and are the primary cause of poliomyelitis [1]. Wild polioviruses (WPV) are on the verge of eradication but in 2020 cases increased to 140 globally [2]. Acute flaccid paralysis (AFP) surveillance remains one of the major tools to monitor for PV and target immunization campaigns [3]. The Global Polio Laboratory Network (GPLN) consists of 145 laboratories in the six World Health Organization (WHO) regions that all follow established guidelines. In the network, laboratories process AFP surveillance stools and perform virus isolation in a combination of two cell lines [4]. Inoculation follows a standard algorithm and results in a sensitive, relatively simple method, which produces high virus titers for subsequent molecular analysis [5]. Virus isolates with cytopathic effect (CPE) are screened with a suite of real-time reverse transcription PCR (RT-PCR) assays contained in the intratypic differentiation (ITD) kit before final verification of a subset of isolates by sequencing [6].

Virus isolation is sensitive but can be lengthy (minimum of 10 days), and requires maintaining two cell lines, the L20B (mouse L cells expressing the human PV receptor [PVR; CD155]) and human rhabdomyosarcoma (RD) cell lines [7, 8]. The WHO *Global Action Plan* III aims to minimize handling and isolation of eradicated PV [9]. Development of a safe, fast, robust, and sensitive cell-culture independent method (direct detection) is a major objective of the Global Polio Eradication Initiative (GPEI).

To constitute a feasible alternative, the procedure must be performed in conjunction with AFP surveillance with potentially high throughput in varying resource settings [10]. It should meet or exceed the sensitivity of virus isolation, be easily implemented without the need to acquire expensive equipment and allow the shipping of reagents and essential consumables to any of the participating GPLN laboratories using standard procedures (e.g. ambient or frozen).

Many enteroviruses (EV) attach to human cell-surface molecules of the immunoglobulin superfamily (IgSF) of proteins [11]. One of these IgSFs, CD155 or poliovirus receptor (PVR), binds PV of any serotype [12]. In the past, similar methods have been used to detect PV from cultured virus isolates or stools [13–16]. These methods were designed to detect or directly sequence EV from stools or to screen environmental surveillance samples and identify PV, without considerations of feasibility for a global laboratory network. The previously mentioned precipitation methods using E.coli-derived PVR proteins or magnetic PVR-beads differed from the present study since both used an in-house produced PVR that was not commercially available (Arita, et al., Abbaszadegan et al.) which were inclusion requirements set by the GPLN at the time this study was conceived. In addition, the previous studies excluded programmatically important PV (i.e. WPV1, PV2 and WPV3). The most recent study published by Shaw et al. showed very promising results and the study design aimed at improving sequencing techniques for PV surveillance stools [17].

A capture assay using recombinant C-terminal 6-histidine-tagged human CD155/PVR protein (PVR-His) was investigated and developed for the concentration of PV-PVR complexes and successive RNA extraction. A method that combined PV-specific targeted capture from stools with existing molecular approaches was found to be as sensitive as the existing WHO- accredited virus isolation protocol. This study is a proof-of-concept for culture-independent detection of PV from AFP surveillance stools, which could serve as an alternative method for the detection of polioviruses.

## Materials and methods

### Standard PV isolation from surveillance stools

Stool suspensions were prepared from AFP or contact cases collected between 1999 and 2016 and virus isolated following a GPLN accredited variation of the standard GPLN method as previously described (inoculation volume 50 μL versus 200 μL, use of 24-well plates versus tubes) [4, 5]. Briefly, 24-well plates with 85–90% confluent monolayers of L20B (recombinant murine cells that express human poliovirus receptor [PVR], National Institute for Biologicals Standards and Control [NIBSC], UK) and RD (American Type Culture Collection [ATCC] CCL-136) cells were prepared 48 hours ahead in 500 μL growth media (minimal essential media [MEM] + 10% fetal bovine serum [FBS]). Before inoculation the growth media was removed and 750 μL maintenance media (MEM + 2% FBS) was added to each well. To inoculate, 50 μL of stool suspension were added onto each of the two wells per cell line leaving 1 well space between samples. The plates were incubated for 5 days, checked daily for CPE and any positive samples (3+ or 4+ CPE) were harvested and inoculated in the opposite cell line and incubated for 5 more days or until CPE was observed. For the blind passage, CPE-negative samples (or 1+ or 2+ CPE) were also harvested but inoculated again on the same cell line and incubated for 5 more days or until CPE was observed. After CPE was observed, the algorithm was followed described here [5, 7, 8, 12].

### Screening of poliovirus RNA by intratypic differentiation real-time RT-PCR

Virus RNA was extracted from stool suspensions using the QIAmp viral RNA minikit following the manufacturer’s instructions and eluted in 50 μL AVE buffer (Qiagen, Germantown, MD). The ITD version 5.0 real-time RT-PCR kit contains primer and hydrolysis probe (TaqMan probes) mixes that discriminate among all three PV serotypes (PV1, 2, and 3) and wildtype genotypes 1 (WPV1 assay) and 3 (WPV3-I, and WPV3-II assays discriminating between West Africa [WEAF-B]; and South Asia [SOAS] genotypes). The ITD kits are produced and distributed by the International Reagent Resource (IRR) contracted by the Centers for Disease Control and Prevention (CDC, Atlanta, GA) [6]. Briefly, the real-time RT-PCR runs were performed using 10 μL qScript XLT 1-Step RT-qPCR ToughMix (QuantaBio, Beverly, MA), adding 1 μL primer and probe mix, 1 μL template, and water for a final reaction volume of 20 μL. PCR screening of CPE-positive isolates was performed with 1 μL of clarified virus isolate (any CPE-positive virus isolate) pipetted directly into the PCR mix or with extracted RNA. The PCRs were conducted in a 7500 thermocycler (Applied Biosystems, Waltham, MA). The results of these screening PCR assays were evaluated qualitatively. During the initial experiments, the direct detection method was evaluated with quantitative RT-PCR (RT-qPCR) using standardized RNA transcripts with known copy numbers that are specific for the PV viral protein 1 (VP1, capsid protein 1) described before [6]. Because in the evaluation experiments RNA extracts were from stool specimens with very low poliovirus content (i.e. cycle threshold [C_T_] values approaching the 95% limit of detection [LOD]) of the PCR assays, the standard 1 μL RNA template volume was increased to 5 μL in 20 μL total PCR volume.

### Standardization of reference virus isolates and AFP surveillance stools for assay development

Poliovirus-positive stool suspensions from AFP surveillance and PV-positive reference virus isolates (e.g., NIBSC reference virus) were selected and standardized for assay development. For the reference virus isolates, infectivity was measured by the endpoint dilution method using RD cell monolayers. Ninety-six well plates were monitored for CPE up to 7 days, and 50% endpoint cell culture infectious dose (CCID_50_·/ 100 μL) titers were calculated by the Kärber method [4, 18]. To standardize AFP surveillance stools, viral titers were measured as previously described [19]. Briefly, HeLa cell monolayers (ATCC CCL-2™) were inoculated with limiting dilutions of 100 μL of 10% stool suspensions and incubated for 5 days while monitoring the CPE. Titers of stool suspensions were expressed as log CCID_50_ per 100 μL. Virus content was verified by RT-qPCR using one of the PCR assays contained in the ITD suite with standardized RNA transcripts (e.g. EV/Sabin assay for quantification of Sabin 1, or WPV1 assays for WPV1).

### Measurement of viral RNA after nuclease treatment

To determine the contribution of free PV RNA in stool suspensions to the overall RNA content measured by RT-qPCR, 130 μL PV2-positive stool suspension (Sabin-like 2, 10^5.88^ CCID_50_ /100 μL) and 100 μL virus reference isolate (Sabin 1, 10^6.2^ CCID_50_) was incubated with 250 units of Benzonase (MilliporeSigma, St. Louis, MO) and 20 units RNAse ONE (Promega, Madison, WI) for 2 h at 37°C, each. Samples were spiked with a PV VP1 RNA transcript derived from a WPV3 virus at a concentration of 10^6^ copies·μL^-1^ as a positive control for the nuclease activity. The same specimens (baseline samples) were processed simultaneously without nucleases. Following the RNA extraction, 5 μL template RNA were assayed by RT-qPCR (ITD kit) with PV VP1 RNA transcripts (Sabin 1, Sabin 2, or WPV3).

### Development of PV enrichment by PVR-His protein capture

Recombinant human CD155/PVR-His-tagged (PVR-His) protein was prepared at a concentration of 100 μg/ml following manufacturer’s instructions (R&D systems, Minneapolis, MN). The PVR-His protein was sourced from a mouse myeloma cell line; the product is the commercial human CD155/PVR protein tagged with a C-terminal 6X-His tag and accession number AAH15542. PVR-capture enrichment was initially evaluated by adding soluble PVR-His protein (7 ng, 14 ng, 70 ng, 0.1 μg, and 1 μg) to standardized Sabin-like 1 positive virus isolate (10^5^ CCID_50_·0.1 mL^-1^). The PV/PVR complex was incubated with 10% nickel-nitrilotriacetic acid (Ni-NTA) agarose (Qiagen) and RNA extracted from 140 μL of pellet after resuspension in minimal essential medium (MEM, Lonza, Walkersville, MD). The lowest concentrations (7–70 ng) of CD155/PVR-His-tagged protein were ineffective, yielding no detectable Sabin 1 RNA, and the two highest PVR-His protein concentrations (0.1, 1 μg) were utilized for all following experiments.

A total of 0.1 μg and 1 μg PVR-His protein was added to 140 μL of virus isolate (standardized Sabin 1). The virus isolate/PVR-His mix was incubated for 2 hours at room temperature rotating on a 360° stand at 18 rpm to allow for virus and PVR-His binding. Virus was captured using Ni-NTA agarose solution (10% v/v) and then incubated at room temperature for 60 min at 18 rpm. After incubation the virus-agarose aggregates were pelleted in a tabletop microfuge at 6800 x *g* and the supernatant removed without disturbing the pellet. The pellet was resuspended in 140 μL Tris/EDTA 0.01M buffer (0.01M TE buffer, Sigma, St. Louis, MO). Viral RNA was extracted from 140 μL of the resuspended pellet and from baseline controls (virus isolate or stool suspension without addition of PVR-His). Viral RNA concentration was measured by RT-qPCR with 1 μL or 10 μL template RNA, and water volumes were adjusted for a final reaction volume to 20 μL. The variation in the volume of template RNA used in the RT-qPCR is due to the sensitivity of the specific PCR assay, i.e. specimens that were close to the limit of detection of the PCR assay 10 μL template were used. Fold-change was calculated using RNA copy numbers of untreated samples and treated samples. The PVR-His capture experiment was repeated using 140 μL standardized stool suspension (Sabin-like 1 10^2.91^ CCID_50_ / 100 μL) instead of virus isolate since this would be the actual specimen type if this method was adopted. The optimization of the PV/PVR-His binding step was empirically determined by adding 140 μL of polyethylene glycol 6000 (PEG6000) in 0.01M TE buffer at concentrations of 7.5% and 15% w/v (to induce a crowding effect) and by adding 0.9% w/v sodium chloride (to aid in precipitation, experiment 3) [20]. Additionally, the method was optimized by testing two concentrations of Ni-NTA agarose solution (10% and 100% v/v) were compared and two incubation times, 2 and 16 h (S3 Table, experiment 4).

Poliovirus nanoparticle capture was initially piloted to determine feasibility. The procedure was described by Minetaro Arita and colleagues from the National Institute of Infectious Diseases of Japan [14]. PVR-sensitized magnetic nanoparticles were kindly provided for preliminary experiments [14]. When 102 pre-titered stools were tested with a low amount of magnetic PVR-labeled nanoparticles (1 μL per sample) compared to RNA recovered from extractions only, a slight but insignificant difference was observed (S1 Fig). The design of this early study was lacking several factors i) only a small number of Sabin 2 positive stools from a clinical trial were tested (missing programmatically important PV stools, i.e. WPV1, VDPV stools), ii) lack of information about standardization of nanoparticles across lots, iii) there was no parallel comparison to poliovirus isolation. Thus, this method was not pursued.

### Detection limits of standard poliovirus isolation

Limiting dilutions of PV-positive stool suspensions were made in 10^0.5^ log steps with PV-negative stool and stored at -80°C. Polioviruses from diluted stool suspensions were isolated following the variation of the virus isolation algorithm [5]. For each stool (n = 4), six dilution steps were tested in the virus isolation procedure using 24-well plates and 50 μL inoculation volume with two wells per cell line (L20B and RD). The absence of EV and PV RNA in the PV-negative stool specimen was verified by assaying 5 μL extracted RNA using the real-time RT-PCR assays contained in the ITD kit. The PV-negative diluent stool suspension was inoculated simultaneously to monitor cell cytotoxicity. The highest dilution that resulted in CPE-positive wells in RD or L20B cells was recorded (more than 80% CPE). For the direct detection method using PVR-His, RNA was extracted from 140 μL of technical replicates of each dilution step followed by assaying 10 μL template RNA twice per RT-qPCR.

### Parallel testing of PVR-His capture and virus isolation

The PVR-His capture method was evaluated empirically with 182 PV-positive stool specimens collected from March 2008 to June 2014. Serotype and genotype information for each stool (virus isolation, ITD real-time RT-PCR assays, or VP1 sequencing results) was collated from CDC database records (Table 1). All three PV serotypes were represented; the majority were WPV1 (*n* = 79), followed by Sabin-like 1 and PV2. Stools stored longer than 10 years were excluded due to the potential impact on the integrity of the virus particles and impact on viability which in turn would affect the virus isolation. There was limited availability of WPV3- and WPV2-positive stools due to either long-term storage, failed virus isolation attempts, or efforts to contain WPV by destruction. Specimens with homotypic (presence of different genotypes but same serotype) and heterotypic mixtures (presence of different serotypes) were included as well. The stool specimens were not tested for enteroviruses that were not polioviruses.

**Table 1:** Composition of stool specimen collection used for parallel testing by poliovirus serotype and genotype determined prior by virus isolation, ITD and sequencing.

Serotype^a^	Genotype^b^	No. (*n* = 182)	Mixtures^c^ (*n* = 16)
PV1	Sabin-like	32	
	WPV1 WEAF-B1 or SOAS	79	
	Homotypic mixture	7	7 (SL1/WPV1)
	Heterotypic mixture	8	3 (SL1/SL3)
			1 (SL1/PV2)
			1 (WPV1/PV2)
			3 (SL1/WPV3)
PV2	Sabin-like/VDPV	32	
	Heterotypic mixture	1	1 (PV2/SL3)
PV3	Sabin-like/VDPV	19	
	WPV3 AFR or SOAS	4	

^a^ PV1 through 3, poliovirus serotypes 1 through 3.

^b^ SL, Sabin-like poliovirus (number denotes serotype); SOAS, South Asia genotype; VDPV, vaccine-derived poliovirus; WEAF-B1, West Africa B1 genotype; WPV3 AFR, wild poliovirus 3 WEAF-B genotype; WPV3 SOAS, wild poliovirus 3 South Asia genotype.

^c^ Number of specimens of the mixtures is included in total count.

Stool suspensions were archived in two 1 mL aliquots and stored at -80°C until further use. Following GPLN accredited variation of the standard WHO GPLN PV isolation procedures, two wells of a 24-well plate were inoculated with 50 μL onto both cell lines (total inoculation volume 100 μl on each cell line) and CPE observed for at least 5 days (method described earlier). Only CPE-positive isolates were screened using 1 μL clarified cell culture supernatant directly in the ITD suite of real-time RT-PCR assays [4–6]. CPE-negative samples were scored negative for virus isolation and not tested in real-time RT-PCR.

For testing the stool specimens by direct detection, the optimized procedure was used as described: 1 μg PVR-His protein was added to 500 μL of stool suspension and mixed with 500 μL 7.5% PEG6000/0.01M TE buffer. The virus/PVR-His pellet was captured using Ni-NTA agarose solution (10% v/v) and then incubated at room temperature for 60 min at 18 rpm. After incubation the virus-agarose aggregates were pelleted in a tabletop microfuge at 6800 x *g* and the supernatant removed without disturbing the pellet as described in the Materials and Methods section (Parallel testing of PVR-His capture and virus isolation). Viral RNA was extracted from 140 μL resuspended PV/PVR-His pellet. Each of the six real-time RT-PCRs contained in the ITD suite of assays were tested with 5 μL template RNA in 20 μL total PCR volume. The C_T_ values for each assay were recorded, when applicable, for each specimen. The PCR screening results were scored following the ITD 5.0 algorithm with the exception of “invalid” results, since they are uncommon in the standard ITD 5.0 algorithm [6]. An invalid result is defined as an illogical result (e.g., samples that are positive for either PanEV or PanPV (but not both) and positive in one of the poliovirus-specific assays). All PCR runs and results were checked for quality and accuracy by a scientist not involved in the parallel study evaluation.

### Statistical analysis

Data analysis and statistical analysis was performed in R with the gmodels package and the stats package version 3.5.3 [21]. The Shapiro-Wilk normality test and the Student’s *t*-test were applied to the magnetic PVR nanoparticles data. Clinical sensitivity, specificity, positive predictive value (PPV), and negative predictive value (NPV) were assessed in 2 x 2 contingency tables inclusive of all tested specimens for the parallel study. Briefly, the proportion of specimens that actually are PV-positive correctly identified by the test (sensitivity), proportion of specimens that are PV-negative and that are correctly identified by the test (specificity); proportion of specimens with a positive test results who actually are PV-positive (PPV); and proportion of specimens with a negative test result who are PV-negative (NPV) [22]. McNemar’s *Χ*^2^ test of independence with continuity correction was applied for significance testing. The assay-by-assay statistics for the ITD PCRs included any positive stool specimen in either of the two methods. Both WPV3-I and WPV3-II real-time RT-PCR assay results showed 100% concordance between direct detection and virus isolation, thus the *Χ*^2^ test of independence could not be applied. Specimens negative by all methods were removed from the analysis.

### Ethical considerations

CDC’s internal program for Human Subjects Research Determination deemed that this study is categorized as public health nonresearch and that human subject regulations did not apply.

## Results

### Free PV RNA in stools and virus isolates does not significantly contribute to the total PV RNA content

Free viral RNA, unassociated with poliovirus particles in stool specimens, could potentially contribute to the total RNA content measured by PCR when testing the enrichment method against RNA extraction [23, 24]. Because the PVR-His captures only intact PV particles, free viral RNA would not be detected, thereby reducing the sensitivity of the procedure. To investigate this hypothesis, two separate experiments were conducted; Sabin 1-positive virus isolates, and Sabin 2-positive stool suspensions were incubated in the presence or absence of nucleases. RNA was extracted and the PV quantity determined by RT-qPCRs targeting specifically Sabin 1, Sabin 2 and the WPV3 RNA spiked control. The quantity of WPV3-specific RNA showed a reduction from 1.8-E4 WPV3 copies/ul (SD 2.3E02) to 12 WPV3 copies/ul (SD 4.2) when treated with nucleases. There were no significant differences in RNA copy numbers after RNA depletion in nuclease-treated and untreated (no addition of nuclease) Sabin 2-positive stool suspensions and CPE-positive virus isolates (Sabin 1) (*P* >0.05, S1 Table). These findings supported the hypothesis that free viral PV RNA was not a confounding measure when PVR-His was used to enrich for PV from stool suspensions and did not contribute to the overall RNA content.

### Soluble His-tagged PVR improved detection of poliovirus from stools

**Fig 1:**
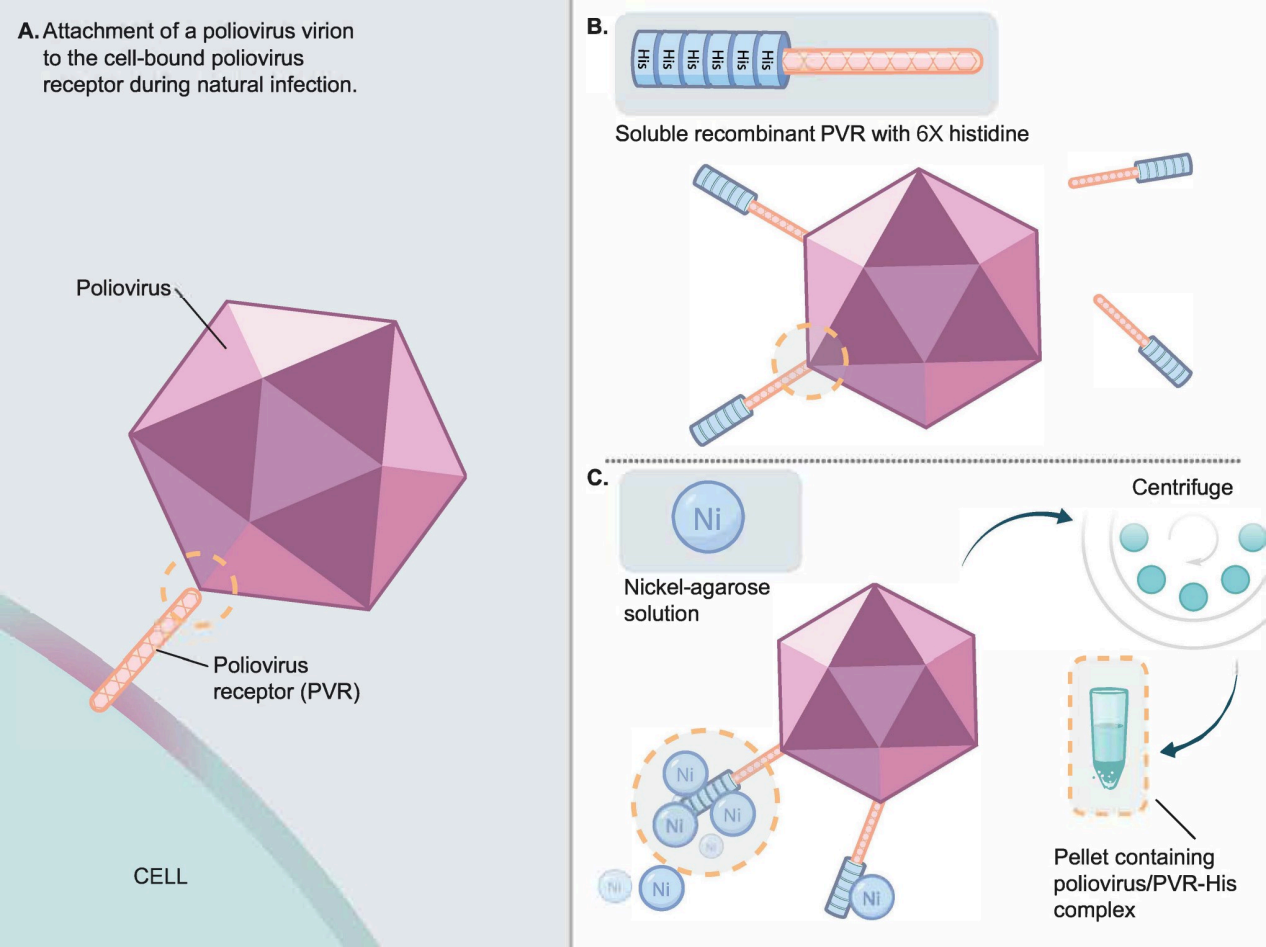
Schematic of poliovirus cellular attachment and the principle of soluble recombinant PVR-His enrichment. **(A)** Poliovirus attaches to cell bound PVR during natural infection. **(B)** Binding of soluble recombinant PVR-histidine and poliovirus. **(C)** Capture of PVR-histidine with nickel-agarose solution and pelleting of the PVR-His/poliovirus nickel agarose complex through centrifugation, removal of supernatant, and downstream application.

The poliovirus capture method is summarized schematically in Fig 1. In the first step, recombinant PVR-His (PVR-His) protein is incubated with stools containing PV to form a PV/PVR-His complex (Fig 1B). In the second step, PV/PVR-His complexes are coupled to nickel agarose residues and concentrated by centrifugation (Fig 1C). RNA is extracted from the pelleted PV/PVR-His/nickel agarose complex prior to performing downstream molecular screening assays.

Using either 0.1 or 1 μg of PVR-His protein improved recovery of Sabin 1 from manual RNA extractions from stool suspensions, 3-fold (0.1 μg, *P* < 0.02) and 7-fold (1 μg, *P* < 0.003), respectively, with the 10% Ni-NTA-agarose solution (Table 2). The addition of 100% Ni-NTA-agarose had a negative effect on the downstream RNA extraction with agarose physically blocking the silica membrane of the extraction columns. The addition of 7.5% PEG 6000 to the 1 μg PVR-His protein/TE buffer improved recovery more than 2-fold over the manual RNA extraction without adding PEG or PVR-His protein (Table 3, *P* < 0.02). Samples with 1 μg PVR-His added but no PEG showed a high deviation. Sodium chloride 0.9% (w/v) was added to the 7.5% PEG 6000/TE buffer with no improvement in PV recovery (S2 Table, experiment 3). Extending the PV/PVR-His incubating step from 2 hours to 16 hours while maintaining the optimal buffer conditions did not significantly increase virus recovery (S3 Table, experiment 4).

**Table 2:** Development of the PVR-His protein capture assay using different concentrations of nickel agarose and PVR-His protein. Fold change of mean RNA copy numbers with PVR-His capture (enrichment step) were compared to extracted RNA without PVR-His. *P*-values were calculated by *t*-test. Quantitative RT-PCR was performed using 1 μL template RNA.

Concentration Nickel-NTA-agarose	PVR-His protein concentration (μg)	Fold change^a^	SD^b^	*P-*value
10%	0.1	3.5	460.39	<0.02
	1	7.7	670.55	<0.003
100%	0.1	0.5	33.07	<0.02
	1	0.5	57.82	<0.03

^a^The copy number of RNA extracted with a manual method from stool suspensions served as the baseline (SD = 111.5 copy/ul).

^b^SD: standard deviation from 4 replicates in copy/ μL

**Table 3:** Development of the PVR-His protein capture assay using different concentrations of PVR-His protein and PEG buffer. Fold change of mean RNA copy numbers with PVR-His capture (enrichment step) in PEG buffer were compared to extracted RNA without PVR-His addition. *P*-values were calculated by *t*-test. Quantitative RT-PCR was performed using 10 μL template RNA.

Concentration of PEG 6000^a^	PVR-His protein concentration (μg)	Fold change^b^	SD^c^	*P-*value
0%	0	N/A	57.5	N/A
0%	0.1	0.9	76.5	>0.05 (n.s.)
0%	1	1.9	373.4	>0.05 (n.s.)
7.5%	0.1	1.7	105.45	<0.02
	1	2.4	192.32	0.02
15%	0.1	1.3	110.91	>0.05 (n.s.)
	1	2.1	169.24	0.01

^a^Ni-NTA-agarose was maintained at 10% v/v.

^b^The copy number of RNA extracted with the manual method from stool suspensions served as the baseline (SD = 57.5 copy/ul).

^c^SD: mean copy numbers per ul from 4 replicates.

Optimal results were achieved with 1 μg PVR-His protein, 500 μL stool suspension, and 500 μL 7.5% PEG/TE buffer incubated for 2 hours with rotation at room temperature. The mixture was then incubated with 10% Ni-NTA-agarose rotating for an additional hour. Immediately after incubation, the PV/PVR-His/nickel agarose complex was pelleted at 6,800 x *g* for 2 min in a tabletop centrifuge. The supernatant was discarded, the remaining pellet PVR-His/PV mix was resuspended in additional 120–130 μL untreated stool suspension (added to maximize recovered viral RNA) to achieve a final volume of 140 μL for viral RNA extraction.

### Culture-independent detection of poliovirus was superior to virus isolation for Sabin 1 containing stool specimens

Detection limits of standard virus isolation and the culture-independent PVR-His method were estimated by testing serially diluted PV-positive stools with previously determined virus titers. A Sabin 1-like positive stool (stool 1) was tested down to 10^0.41^ CCID_50_ /100 μL in stool for both methods (Table 4). CPE (Sabin-1) was still detectable at a titer of 10^0.91^ CCID_50_ /100 μL using the PVR-His capture method, whereas the stool was CPE negative in virus isolation below the 10^2.41^ CCID_50_ /100 μL stool titer step (S4 Table). When three WPV1-positive stool specimens (stools 2–4) were isolated in cell culture, CPE was observed with titers as low as 10^1.28^ CCID_50_ /100 μL (stool 3, Table 4). The culture-independent PVR-His capture method detected WPV1 in diluted stools at 10^1.56^ CCID_50_ /100 μL (stool 2) but missed WPV1 RNA in lower dilutions (stools 3–4). The RNA content in the diluted stools specimens was close to the detection limit of the WPV1 RT-PCR assay (95% LOD 100 copies/ μL).

**Table 4:** Comparison of virus isolation and the PVR-His protein capture method on limiting dilutions of PV1-positive stools. The lowest titer of poliovirus by culture and the lowest titer of poliovirus detectable by the PVR-His capture assay is listed.

Specimen	Genotype	Titer (log_10_ CCID_50_ /100 μL)	Titer range^a^ (log_10_ CCID_50_ /100 μL)	Lowest titer isolated/detected (log_10_ CCID_50_ /100 μL)	
				Virus isolation^b^	PVR-His method^c^
stool 1	Sabin 1	2.91	0.41–2.91	2.41	0.91
stool 2	WPV1	3.56	0.06–3.56	1.56	1.56
stool 3	WPV1	3.78	0.28–3.78	1.28	1.78
stool 4	WPV1	3.39	0.89–3.39	2.89	3.39
stool 5^d^	negative	N/A	N/A	N/A	N/A

^a^ For each stool specimen, at least six dilution steps were tested following standard virus isolation.

^b^ Duplicates for each dilution were tested in RD and L20B cells (e.g., stool 1, *n* = 4 observations per dilution step).

^c^ Both, RNA extraction from resuspended PV/PVR-His pellets and RT-PCRs testing were performed in duplicate.

^d^ Stool suspension that was CPE-negative was used as diluent (PV-negative after virus isolation and confirmed by RT-PCR screening in PanPV and Quadruplex/EV assays).

### PVR method was more sensitive than PV isolation in parallel testing

A total of 182 stools were tested by PVR-His enrichment and RNA extraction in parallel with standard virus isolation according to WHO protocols, followed by screening in the ITD 5.0 real-time RT-PCR assays. The PVR-His method showed a high sensitivity of 96.2% compared to virus isolation (*P* <0.01). Virus isolation failed to detect poliovirus in 51 samples, whereas 18 were missed by the PVR-His method (Table 5). For samples that were missed by both methods (*n* = 13), ten stools were PCR-positive for Enterovirus RNA only (EV specific RT-PCR assay) in the PVR-His method but could not be isolated using the standard GPLN isolation algorithm. This was likely due to loss of virus viability during years of storage, combined with low virus titers in the inoculum and might also be caused by the fact that the virus isolation algorithm favors polioviruses. Both the predictive-value positives (76.8%) and the predictive-value negatives (72.2%) were high with the PVR-His capture method. Virus isolation had a higher false negative rate of 23.2% [38/164] compared to direct detection (3.8% [5/131]).

**Table 5:** Two-by-two table comparison of poliovirus detection in the culture-independent detection method (PVR-His capture) followed by RNA extraction and standard virus isolation. Results from a total of 182 stool specimens are shown that were selected on the results from previous testing for poliovirus in virus isolation, ITD PCR, and VP1 sequencing (Table 1).

	Virus isolation		
PVR-His capture^a^	Positive	Negative	Total^b^
Positive	126	38	164
Negative	5	13	18
Total	131	51	182

^a^Any poliovirus detected in ITD suite of PCR assays were scored positive (including invalid and indeterminate results). Samples that were negative in all ITD assays were scored negative for poliovirus.

^b^Sensitivity: 96.2%, specificity: 25.5%, (Predictive value positive [PVP] 76.8%, predictive value negative [PVN]: 72.2%); McNemar *χ*^*2*^ with continuity correction *P* = 1.1E-06.

**Fig 2:**
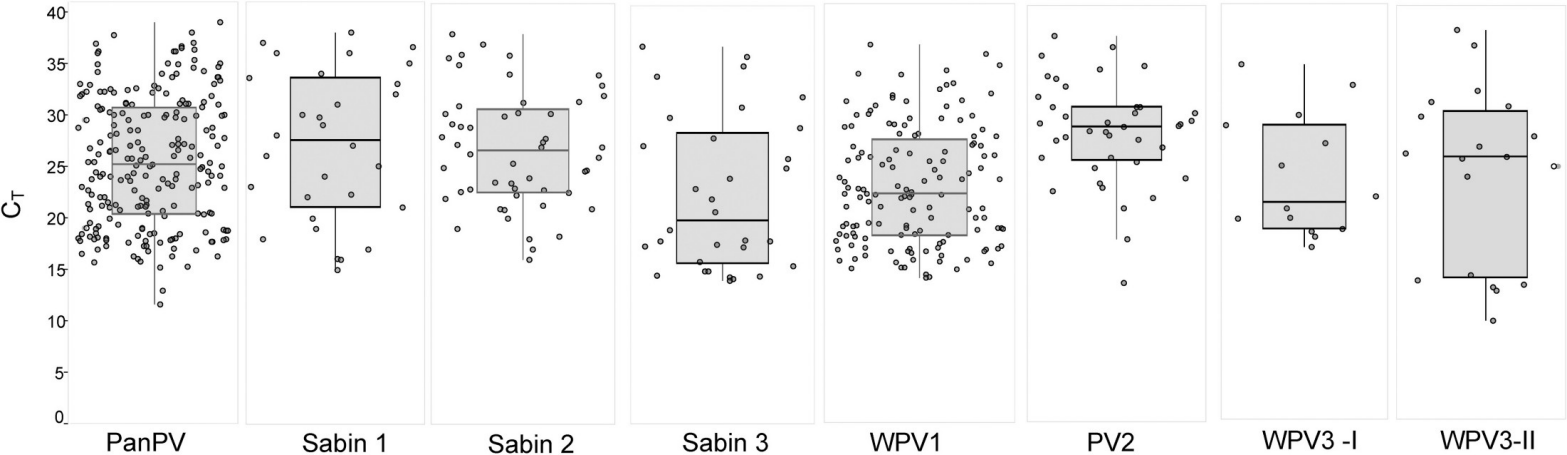
Parallel testing assay-by-assay results for PV-positive stools. Poliovirus RNA was extracted after PVR-His enrichment and screened in ITD real-time RT-PCR for all samples. C_T_ values are shown by ITD assay and when positive in PVR-His enrichment/RNA extraction but negative in virus isolation.

To determine if the concentration or random error contributed to the results, C_T_ value differences were analyzed between positive stools in both methods and positive stools only in the culture-independent method. For the stool suspensions that were positive in both methods, median C_T_ values were below 30 for any of the 8 targets in the ITD assays, indicating both methods were able to detect poliovirus from stools with higher viral titers (Figs 2 and S1). For samples that were only positive by the PVR-His capture, C_T_ values ranged between 28 and 38 for most of the real-time RT-PCRs (S2 Fig). Samples with lower virus RNA content (C_T_s above 30) were still detected in the culture-independent method depending on the LOD of the specific assay, *e*.*g*., PanPV assay ranges between 1 CCID_50_·μl^-1^ (PV Sabin 2 and 3) and 100 CCID_50_·μl^-1^ (WPV1) [6]. Thirty-one PV mixtures were detected by the PVR-His capture method, and 14 mixtures by virus isolation (S5 Table). The PVR-His capture method outperformed the standard virus isolation method in the assay-by-assay results comparing all 171 positive stools for each assay (only PCR-positive RNA specimens with Ct values were included for statistical analysis; S6 Table). In the PanPV, Sabin 1, Sabin 2, and Sabin 3 real-time RT-PCR assays, the number of detected PV was significantly higher (e.g., PanPV *P* <0.001) for PVR-His capture method. When the detection rate was compared in stools that were previously confirmed by virus isolation, PCR, and sequencing to be WPV1-positive (n = 87; including 8 mixtures); PVR-capture missed 15 WPV1-positive, and virus isolation missed seven WPV1-positive stools, although the difference was not significant (*P* >0.05) (S6 Table). These 15 undetected WPV1-positive samples were positive in the EV and PanPV PCR assays and were identified as “indeterminate PV” through the ITD screening; they would be sequenced as part of the current PV detection algorithm. From 164 PVR-capture positive specimens, 13 were “invalid” after PCR screening, and would be flagged for PV to undergo virus isolation or further analysis. Overall, when considering all PCR assays, the PVR-His tag method was significantly more sensitive (*P* <0.05) than virus isolation in the detection of poliovirus (Tables 5 and S6).

## Discussion

Cell culture-independent detection, known as direct detection of polioviruses, from AFP surveillance stools is one of the immediate goals of the GPLN. Adopting a culture-independent method for AFP surveillance stools would greatly reduce the number of labs that need to isolate poliovirus, while providing rapid results. The His-tagged PVR method was developed and tested for the selective capture and enrichment of PV particles from stool specimens and extracted RNA recovery estimated with RT-qPCR assays contained in the ITD suite [4, 6]. The method using soluble recombinant PVR-His protein was optimized for PV-containing stools. Using pre-titered stools, the LOD was estimated and compared to the standard virus isolation method. The optimized PVR-His capture technique was tested with 182 surveillance stools and was found to be non-inferior to the GPLN-accepted variation of the virus isolation method.

When PV-containing stool specimens were treated with nucleases to deplete free RNA, we detected no significant contribution of free PV-RNAs to the quantities of total PV-RNA observed. This finding was essential in the beginning of the method development because receptor-based assays target intact poliovirus particles. Any free PV RNA would be removed in the washing procedures following the initial poliovirus capture. We hypothesize that when preparing stools in a chloroform suspension, like that used in the GPLN, little free or unpacked PV RNA remains intact in the sample.

After infection, PV replicates in the gut, and PV-positive stools may contain a wide-ranging concentration of PV [19]. We were specifically interested in stool specimens with low virus amounts that might be missed by virus isolation or in molecular assays (approaching the lower limit of PCR sensitivity). To increase sensitivity over manual RNA extraction, the soluble PVR-His protein was applied to PV-containing stools and buffers and concentrations optimized (Fig 1).

Additionally, this culture-independent detection assay was compared to the GPLN-accepted variation of the standard virus isolation method. Although virus isolation uses 200 μL for each cell line for inoculation, the CDC polio lab has been using an adapted method using less volume (100 μL each cell line RD and L20B). The polio laboratory is accredited by the GPLN as a global specialized laboratory and has successfully participated in annual proficiency panels in virus isolation showing the ability of the laboratory to detect polioviruses from surveillance specimens. This comparison allowed us to estimate the lower limit of detection for the PVR-His culture-independent approach. The PVR-His capture technique showed higher sensitivity with Sabin 1, but similar or worse LOD with three WPV1-positive stools compared to virus isolation. For the virus isolates, it is unlikely that other EVs present in the sample would have contributed to the total CPE that could not be distinguished from WPV1-derived CPE by culture alone, since virus isolation favors the growth of polioviruses in L20B cells. These CCID_50_ estimates could not be verified, as the titration method’s detection limit is at 10^2.75^ CCID_50_ /100 μL stool [25]. Of note the extracted RNA from WPV1-positive stools was tested in the specific WPV1 RT-PCR assay which is less sensitive (95% LOD 100 copies / μL) than the Sabin 1 assay (95% LOD 1 copy/ μL; component of the EV/Sabin RT-PCR assay).

When we tested the PVR-His tagged capture with 182 stools from the CDC collection, it outperformed the virus isolation method with higher numbers of PV positive samples through PCR- screening than CPE-positive isolates. Thirteen stools were now PV-negative in both methods, while ten stools were PCR-positive for Enterovirus RNA only (EV specific assay after PVR-His method). From the 171 PV-positive stools (any poliovirus detected in either method by screening PCR assay), virus isolation missed 38, including seven WPV1-positive stools. The collection was comprised of programmatically relevant WPV1 (presently endemic in two countries and recently eradicated in Africa) and PV2, as well as PV3-positive stool specimens. One possible explanation for false-negative outcomes might be the storage time of stools or repeated freezing and thawing affecting PV integrity and viability detrimental to the virus isolation procedure. Unlike virus isolation, the PVR-His capture assay does not completely depend on viability, but rather on overall RNA content. The benefit is compounded by the resuspension of the pellet in clarified stool suspension, increasing viral RNA recovery. This advantage over the standard method was supported by the higher overall sensitivity measured by PCR from specimens that were missed by virus isolation but detected through PVR-His capture. Overall, direct detection with soluble, recombinant PVR-His protein captured PV particles, and when combined with column-based RNA extraction, performed non-inferior to virus isolation.

One of the limitations in this study was that the assay worked well in our laboratory with scientists that are highly trained in molecular methods; other WHO GPLN labs may not have experienced personnel available. Current virus isolation protocols are easy to follow and have been used for decades with a current yearly throughput of up to 200,000 specimens globally [26]. The culture-independent detection is easy to perform in any molecular laboratory and uses already existing lab equipment such as a microfuge and routine techniques such as RNA extraction. It does require the purchase of an inexpensive rotator and the preparation of specialized reagents by lab scientists (e.g., PEG/TE buffer). The buffer and assay components are widely available from commercial vendors and preparation methods are easy to follow with the added benefit of faster results.

The direct detection method using PVR-His presents challenges to the implementation in the GPEI, such as finding a process control, to provide quality measures for participating laboratories and the costs of the provision and distribution of the recombinant PVR-His protein to the entire network. The method would have to be piloted in select laboratories as part of a validation with AFP specimens. It would increase the cost per sample, since RNA would have to be extracted and followed by PV detection in RT-PCR for each specimen, whereas a large proportion of specimen turns out negative (95%, 190,000 specimens/annually) and gets discarded in the gold standard virus isolation method. Another challenge is that the testing algorithm would need to be revised for culture-independent PV detection reducing the number of simultaneously run PCR assays. Invalid, indeterminate, or false-positive results in ITD assays would yield higher workloads for sequencing and regional reference laboratories since per proposed algorithm those specimens would be referred to them for further characterization (including virus isolation). In turn, screening samples with a direct detection method would minimize the number of samples that would eventually need to go through virus isolation (taking up to 2 weeks), reducing the amount of time to recognize outbreaks.

Future work will compare automated and manual RNA extraction systems, which would further increase the turnaround time as well as scale. There are challenges in implementation of automated RNA extraction systems worldwide because of the cost for the initial purchase and the associated need for regular maintenance of instruments, but this challenge might be alleviated by the investments in national surveillance systems infrastructure amidst the COVID-19 pandemic (SARS-CoV-2 also is positive-sense single stranded RNA virus) [27]. The PVR-His method may be flexible enough to be combined with automated RNA extraction but needs further validation. The availability of PVR-His protein from international vendors is the ideal scenario for the GPLN program but would require prior compatibility testing to establish guidelines for protein binding efficiency. The method also might be applicable to rescue PV particles from environmental surveillance concentrates.

A receptor-based enrichment procedure could be used as a targeted capture procedure for other enteroviruses, such as Coxsackie A viruses 13, 15, 18, 20 and 21 are known to recognize the ICAM-1, or EV71, using recombinant receptor SCARB2 [11, 12, 28, 29]. The underlying principle of this PV/PVR capture method has the potential to be useful for targeted capture of other pathogenic human viruses present in human-derived specimens outside of the GPLN.

## Supporting information

S1 Fig Sensitivity of study stools by assay. The ITD assay results are shown as C_T_ values for stools that were positive in virus isolation and positive in the PVR-His enrichment method (n = 131) [PanPV (any poliovirus), PV2 (any serotype 2), WPV1 (any wild poliovirus 1), WPV3-I (wild poliovirus 3—WEAF-B genotype), WPV3-II (wild poliovirus 3—South Asia genotype)].
(PDF)

S2 Fig Sensitivity of study stools by assay. The ITD assay results are shown as C_T_ values for stools that were negative in virus isolation and positive in the PVR-His enrichment method (n = 38) [PanPV (any poliovirus), PV2 (any serotype 2), WPV1 (any wild poliovirus 1)].
(PDF)

S1 Table Average quantity of WPV3 RNA copies per microliter for qRT-PCR (WPV3 AFR assay). (PDF)

S2 Table Development of the PVR-His capture assay with or without saline. Fold change of mean RNA copy numbers with PVR-His protein capture (treated) were compared to untreated samples (RNA extracted without capture) and *p*-values calculated by t-test. PCR was performed using 10 μL of template RNA in 20 μL final reaction volume (Exp. 3).
(PDF)

S3 Table Development of the His-PVR capture assay with varying incubation times. Fold change of mean RNA copy numbers with PVR-His protein capture (treated) were compared to untreated samples (RNA extracted without capture) and *p*-values calculated by t-test. PCR was performed using 1 μL or 10 μL of template RNA in 20 μL final reaction volume (Exp. 4).
(PDF)

S4 Table Two-by-two table for the subset of stools containing mixtures (*n* = 34) that were assayed in parallel by virus isolation (standard method) and by the new PVR-His protein capture method. (PDF)

S5 Table Stool suspensions diluted and tested side by side with PVR-His tag enrichment and RNA extraction or only RNA extraction and PV content determined by qPCR (Sabin 1 target). (PDF)

S6 Table Statistical analysis performed in R. Data were generated comparing 171 stools that were positive in both PVR-His capture (PVR) and standard virus isolation (VI). Chi-square and post-hoc test results are included as well.
(PDF)
