# Supplementary material for: Direct detection of polioviruses using a recombinant poliovirus receptor
Source: PLoS One. 2021 Nov 2;16(11):e0259099. doi: 10.1371/journal.pone.0259099 (PMC8562806; doi:10.1371/journal.pone.0259099)
Supplement: S1 Fig — The ITD assay results are shown as CT values for stools that were positive in virus isolation and positive in the PVR-His enrichment method (n = 131) [PanPV (any poliovirus), PV2 (any serotype 2), WPV1 (any wild poliovirus 1), WPV3-I (wild poliovirus 3—WEAF-B genotype), WPV3-II (wild poliovirus 3—South Asia genotype)]. (PDF) [file pone.0259099.s001.pdf]

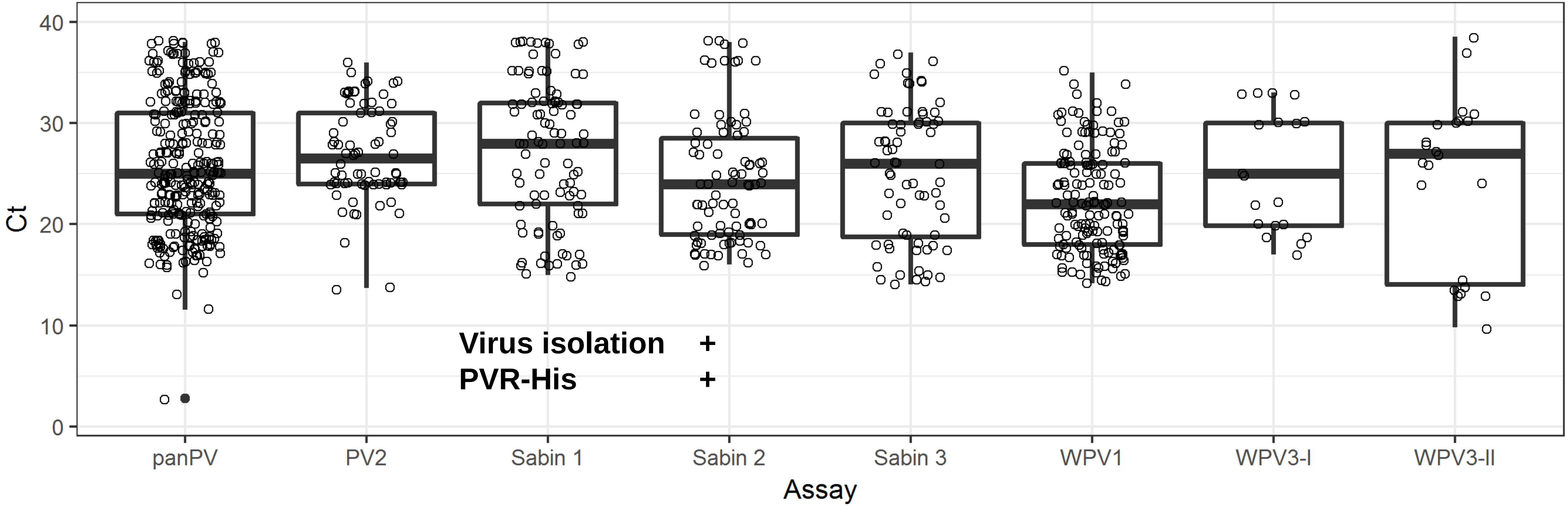

S1 Figure. The ITD assay results are shown as C_t values for samples that were positive in virus isolation and positive in the PVR-His enrichment method (n=131). [panPV (any poliovirus), PV2 (any serotype 2), WPV1 (any wild poliovirus 1), WPV3-I (wild poliovirus 3 - African genotype), WPV3-II (South-Asia genotype)]
