# Supplementary material for: Direct detection of polioviruses using a recombinant poliovirus receptor
Source: PLoS One. 2021 Nov 2;16(11):e0259099. doi: 10.1371/journal.pone.0259099 (PMC8562806; doi:10.1371/journal.pone.0259099)
Supplement: S2 Fig — The ITD assay results are shown as CT values for stools that were negative in virus isolation and positive in the PVR-His enrichment method (n = 38) [PanPV (any poliovirus), PV2 (any serotype 2), WPV1 (any wild poliovirus 1)]. (PDF) [file pone.0259099.s002.pdf]

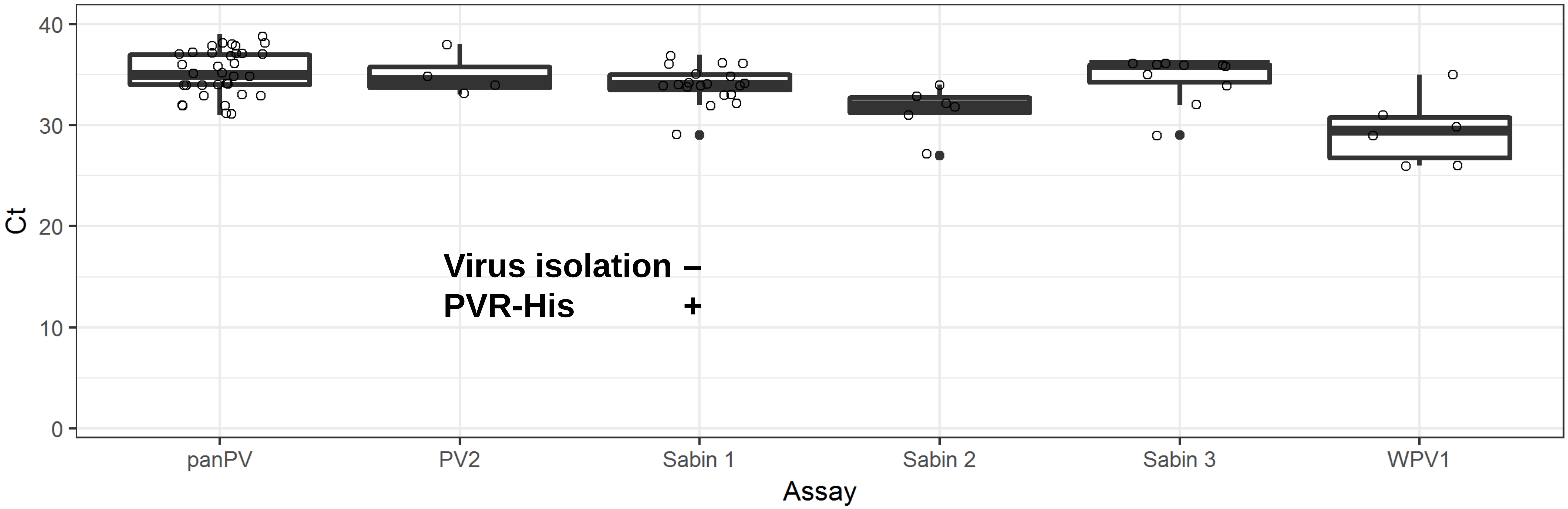

S2 Figure. The ITD assay results are shown as C_t values for samples that were negative in virus isolation and positive in the PVR-His enrichment method (n=38)
[panPV (any poliovirus), PV2 (any serotype 2), WPV1 (any wild poliovirus 1)]
