# Supplementary material for: Direct detection of polioviruses using a recombinant poliovirus receptor
Source: PLoS One. 2021 Nov 2;16(11):e0259099. doi: 10.1371/journal.pone.0259099 (PMC8562806; doi:10.1371/journal.pone.0259099)
Supplement: S1 Table — (PDF) [file pone.0259099.s003.pdf]

S1 Table Average quantity of WPV3 RNA copies per microliter for qRT-PCR (WPV3 AFR assay)

Treatment	Mean Copies/ ul	Mean SD	Mean (biol. Replicates 1 and 2)	SD
Nuclease treated (1)	9.15	5.59	12.08	4.14
Nuclease treated (2)	15.01	1.01		
Not treated (1)	16879.97	7112.75	18671.69	2533.8
Not treated (2)	20463.40	1568.37		
