# Supplementary material for: Direct detection of polioviruses using a recombinant poliovirus receptor
Source: PLoS One. 2021 Nov 2;16(11):e0259099. doi: 10.1371/journal.pone.0259099 (PMC8562806; doi:10.1371/journal.pone.0259099)
Supplement: S2 Table — Fold change of mean RNA copy numbers with PVR-His protein capture (treated) were compared to untreated samples (RNA extracted without capture) and p-values calculated by t-test. PCR was performed using 10 μL of template RNA in 20 μL final reaction volume (Exp. 3). (PDF) [file pone.0259099.s004.pdf]

S2 Table Development of the His-PVR capture assay (Experiment 3). Fold change of mean RNA copy numbers with His-PVR protein capture (treated) were compared to untreated samples (RNA extracted without capture) and *P*-values calculated by t-test. PCR was performed using 10 µL of template RNA in 20 µL final reaction volume.

Conditions tested	PVR-His protein concentration (µg)	Fold change	*P*-value
**Sodium chloride v/v**			
No saline	0.1	1.9	<0.02
	1	2.2	<0.01
0.9% saline	0.1	n/a*	n/a
	1	2.7	>0.05 (n.s.)
0.9% NaCl vs. no saline	0.1	1.2	>0.05 (n.s.)

[#]Ni-NTA-agarose was maintained at 10% v/v, and PEG 6000 7.5% v/v in 0.01M TE buffer for experiment 3.
*n/a: RNA not recovered in experiment (no C_T values) or PCR was inhibited by high salt content that was not removed during RNA extraction
