# Supplementary material for: Direct detection of polioviruses using a recombinant poliovirus receptor
Source: PLoS One. 2021 Nov 2;16(11):e0259099. doi: 10.1371/journal.pone.0259099 (PMC8562806; doi:10.1371/journal.pone.0259099)
Supplement: S3 Table — Fold change of mean RNA copy numbers with PVR-His protein capture (treated) were compared to untreated samples (RNA extracted without capture) and p-values calculated by t-test. PCR was performed using 1 μL or 10 μL of template RNA in 20 μL final reaction volume (Exp. 4). (PDF) [file pone.0259099.s005.pdf]

S3 Table Development of the His-PVR capture assay (Experiment 4). Fold change of mean RNA copy numbers with His-PVR protein capture (treated) were compared to untreated samples (no His-PVR capture) and *P*-values calculated by t-test. PCR was performed using 1 µL or 10 µL of template RNA in 20 µL final reaction volume.

**Conditions tested#**	**PCR template (µL)**	**Fold change (treated vs controls)**	***P*-value**
**Incubation duration (h)**			
2	1	2.9	<0.03
	10	1.7	>0.05 (n.s.)
16	1	2.7	>0.05 (n.s.)
	10	2.2	>0.05 (n.s.)
**Fold change of 2 h vs 16 h**	1		0.88 (n.s.)
	10		0.86 (n.s.)

#0.1 µg PVR-His protein in 7.5% PEG 6000 in 0.01M TE buffer, Ni-NTA-agarose 10% v/v.

.
