# Supplementary material for: Direct detection of polioviruses using a recombinant poliovirus receptor
Source: PLoS One. 2021 Nov 2;16(11):e0259099. doi: 10.1371/journal.pone.0259099 (PMC8562806; doi:10.1371/journal.pone.0259099)
Supplement: S4 Table — (PDF) [file pone.0259099.s006.pdf]

S4 Table Stool suspensions diluted and tested side by side with PVR-His tag enrichment and RNA extraction or only RNA extraction and PV content determined by qPCR (Sabin 1 target).

log titer	Sample No	Treatment	Value_1	Value_2	Value_3	Value_4	Average	SD
2.91	001-2.91	2.91 log Qiagen extraction	1266.40	649.94	617.88	684.99	805	308.95
2.41	002-2.41	2.41 log Qiagen extraction	63.54	54.39	145.61	192.73	114	66.58
1.91	003-1.91	1.91 log Qiagen extraction	24.35	3.79	12.39	41.66	21	16.40
1.41	004-1.41	1.41 log Qiagen extraction	2.74	1.69		2.58	2	0.57
0.91	005-0.91	0.91 log Qiagen extraction	0	0	0	0	0	0.00
0.00	006-0.41	0.41 log Qiagen extraction	0	0	0	0	0	0.00
2.91	001-2.91	2.91 log PVR-His tag + Qiagen extraction	1230.18	999.11	1564.78	1375.37	1292	238.73
2.41	002-2.41	2.41 log PVR-His tag + Qiagen extraction	314.48	1448.81	201.03	234.62	550	601.27
1.91	003-1.91	1.91 log PVR-His tag + Qiagen extraction	66.95	72.54	39.96	65.31	61	14.49
1.41	004-1.41	1.41 log PVR-His tag + Qiagen extraction	41.89	33.14	12.55	32.60	30	12.41
0.91	005-0.91	0.91 log PVR-His tag + Qiagen extraction	2.95	4.20	15.38	7.42	7	5.59
0.00	006-0.41	0.41 log PVR-His tag + Qiagen extraction	3.48	0	0	0	1	1.74
N/A	007-neg	negative stool pellet + Qiagen extraction	0	0	0	0	0	0.00
