# Supplementary material for: Direct detection of polioviruses using a recombinant poliovirus receptor
Source: PLoS One. 2021 Nov 2;16(11):e0259099. doi: 10.1371/journal.pone.0259099 (PMC8562806; doi:10.1371/journal.pone.0259099)
Supplement: S5 Table — (PDF) [file pone.0259099.s007.pdf]

S5 Table Two-by-two table for the subset of stools containing mixtures (n = 34) that were assayed in parallel by virus isolation (standard method) and by the new PVR-His protein capture method (direct detection).

	**Virus Isolation**		
**His-PVR capture**[a]	Positive	Negative	Total
Positive	11	20	31
Negative	3	0	3
Total	14	20	34

[a] Any poliovirus detected in ITD suite of PCR assays = positive. Samples scored negative when all ITD assay results were negative
