# Supplementary material for: Direct detection of polioviruses using a recombinant poliovirus receptor
Source: PLoS One. 2021 Nov 2;16(11):e0259099. doi: 10.1371/journal.pone.0259099 (PMC8562806; doi:10.1371/journal.pone.0259099)
Supplement: S6 Table — Data were generated comparing 171 stools that were positive in both PVR-His capture (PVR) and standard virus isolation (VI). Chi-square and post-hoc test results are included as well. (PDF) [file pone.0259099.s008.pdf]

S6 Statistical analysis performed in R. Data were generated comparing 171 stools that were positive in both His-PVR capture (PVR) and standard virus isolation (VI). Chi-square and post-hoc test results are included as well.

```
Cell Contents
------------------------|
                  Count |
Chi-square contribution |
            Row Percent |
         Column Percent |
          Total Percent |
           Std Residual |
 -----------------------|

Pan PV assay: Total Observations in Table:  171
                        | a.2.1_VI.panPV$panPV
a.2.1_PVR.panPV$panPV   | negative  | positive  | Row Total |
------------------------|-----------|-----------|-----------|
               negative |        9  |        8  |       17  |
                        |   6.346   |   1.938   |           |
                        |   52.941% |   47.059% |    9.942% |
                        |   22.500% |    6.107% |           |
                        |    5.263% |    4.678% |           |
                        |  2.519    |   -1.392  |           |
    --------------------|-----------|-----------|-----------|
               positive |       31  |      123  |      154  |
                        |   0.701   |   0.214   |           |
                        |   20.130% |   79.870% |   90.058% |
                        |   77.500% |   93.893% |           |
                        |   18.129% |   71.930% |           |
                        |   -0.837  |   0.462   |           |
    --------------------|-----------|-----------|-----------|
           Column Total |       40  |      131  |      171  |
                        |   23.392% |   76.608% |           |
    --------------------|-----------|-----------|-----------|
McNemar's Chi-squared test : Chi^2 =  13.5641     d.f. =  1     p =  0.0002305526
McNemar's Chi-squared test with continuity correction: Chi^2 =12.41026, d.f. =  1, p
=  0.0004269822; Minimum expected frequency: 3.976608, Cells with Expected Frequency
< 5: 1 of 4 (25%)

Sabin 1 Assay: Total Observations in Table:  171
                        | a.2.1_VI.sabin1$sabin1
a.2.1_PVR.sabin1$sabin1 | negative  | positive  | Row Total |
------------------------|-----------|-----------|-----------|
               negative |      121  |        0  |      121  |
                        |    1.681  |  13.444   |           |
                        |  100.000% |    0.000% |   70.760% |
                        |   79.605% |    0.000% |           |
                        |   70.760% |    0.000% |           |
                        |    1.296  |   -3.667  |           |
```

```
------------------------|-----------|-----------|-----------|
               positive |        31 |        19 |        50 |
                        |     4.067 |    32.536 |           |
                        |   62.000% |   38.000% |   29.240% |
                        |   20.395% |  100.000% |           |
                        |   18.129% |   11.111% |           |
                        |    -2.017 |     5.704 |           |
------------------------|-----------|-----------|-----------|
           Column Total |       152 |        19 |       171 |
                        |   88.889% |   11.111% |           |
------------------------|-----------|-----------|-----------|
McNemar's Chi-squared test : Chi^2 =  31     d.f. =  1     p =  2.580284e-08
McNemar's Chi-squared test with continuity correction : Chi^2 =  29.03226     d.f. =
 1     p =  7.118304e-08 ; Minimum expected frequency: 5.555556

Sabin 2 assay: Total Observations in Table:  171
                        | a.2.1_VI.sabin2$sabin2
a.2.1_PVR.sabin2$sabin2 | negative  | positive  | Row Total |
------------------------|-----------|-----------|-----------|
               negative |       133 |         0 |       133 |
                        |     4.264 |    21.778 |           |
                        |  100.000% |    0.000% |   77.778% |
                        |   93.007% |    0.000% |           |
                        |   77.778% |    0.000% |           |
                        |     2.065 |    -4.667 |           |
------------------------|-----------|-----------|-----------|
               positive |        10 |        28 |        38 |
                        |    14.925 |    76.222 |           |
                        |   26.316% |   73.684% |   22.222% |
                        |    6.993% |  100.000% |           |
                        |    5.848% |   16.374% |           |
                        |    -3.863 |     8.731 |           |
------------------------|-----------|-----------|-----------|
           Column Total |       143 |        28 |       171 |
                        |   83.626% |   16.374% |           |
------------------------|-----------|-----------|-----------|
McNemar's Chi-squared test : Chi^2 =  10     d.f. =  1     p =  0.001565402
McNemar's Chi-squared test with continuity correction: Chi^2 = 8.1, d.f. =  1, p =
0.004426526: Minimum expected frequency: 6.222222

Sabin 3 assay: Total Observations in Table:  171
                        | a.2.1_VI.sabin3$sabin3
a.2.1_PVR.sabin3$sabin3 | negative  | positive  | Row Total |
------------------------|-----------|-----------|-----------|
               negative |       141 |         0 |       141 |
                        |     1.746 |    14.842 |           |
                        |  100.000% |    0.000% |   82.456% |
                        |   92.157% |    0.000% |           |
                        |   82.456% |    0.000% |           |
                        |     1.321 |    -3.853 |           |
```

positive	12	18	30
	8.207	69.758	
	40.000%	60.000%	17.544%
	7.843%	100.000%	
	7.018%	10.526%	
	-2.865	8.352	
Column Total	153	18	171
	89.474%	10.526%	

McNemar's Chi-squared test : Chi^2 = 12   d.f. = 1   p = 0.0005320055
McNemar's Chi-squared test with continuity correction: Chi^2 = 10.08333 d.f. = 1 p = 0.001496164 ; Minimum expected frequency: 3.157895; Cells with Expected Frequency < 5: 1 of 4 (25%)

PV2 assay: Total Observations in Table: 171

	a.2.1_VI.PV2$PV2		
a.2.1_PVR.PV2$PV2	negative	positive	Row Total
negative	138	1	139
	4.074	20.804	
	99.281%	0.719%	81.287%
	96.503%	3.571%	
	80.702%	0.585%	
	2.018	-4.561	
positive	5	27	32
	17.694	90.368	
	15.625%	84.375%	18.713%
	3.497%	96.429%	
	2.924%	15.789%	
	-4.206	9.506	
Column Total	143	28	171
	83.626%	16.374%	

McNemar's Chi-squared test: Chi^2 = 2.666667   d.f. = 1   p = 0.1024704
McNemar's Chi-squared test with continuity correction: Chi^2 = 1.5   d.f. = 1 p = 0.2206714 ; Minimum expected frequency: 5.239766

WPV1 assay: Total Observations in Table: 171

	a.2.1_VI.WPV1$WPV1		
a.2.1_PVR.WPV1$WPV1	negative	positive	Row Total
negative	90	15	105
	15.556	20.390	
	85.714%	14.286%	61.404%
	92.784%	20.270%	
	52.632%	8.772%	

	3.944	-4.516	
positive	7	59	66
	24.747	32.439	
	10.606%	89.394%	38.596%
	7.216%	79.730%	
	4.094%	34.503%	
	-4.975	5.696	
Column Total	97	74	171
	56.725%	43.275%	

McNemar's Chi-squared test : Chi^2 = 2.909091 d.f. = 1 p = 0.08808151
McNemar's Chi-squared test with continuity correction: Chi^2 = 2.227273 d.f. = 1 p = 0.135593 ; Minimum expected frequency: 28.5614

WPV3-A assay: Total Observations in Table: 171

	a.2.1_VI.WPV3A$WPV3A		
a.2.1_PVR.WPV3A$WPV3A	negative	positive	Row Total
negative	165	0	165
	0.211	5.789	
	100.000%	0.000%	96.491%
	100.000%	0.000%	
	96.491%	0.000%	
	0.459	-2.406	
positive	0	6	6
	5.789	159.211	
	0.000%	100.000%	3.509%
	0.000%	100.000%	
	0.000%	3.509%	
	-2.406	12.618	
Column Total	165	6	171
	96.491%	3.509%	

McNemar's Chi-squared test : Chi^2 = NaN d.f. = 1 p = NaN, Minimum expected frequency: 0.2105263; Cells with Expected Frequency < 5: 1 of 4 (25%)

WPV3-S assay: Total Observations in Table: 171

	a.2.1_VI.WPV3S$WPV3S		
a.2.1_PVR.WPV3S$WPV3S	negative	positive	Row Total
negative	165	0	165
	0.211	5.789	
	100.000%	0.000%	96.491%
	100.000%	0.000%	

	96.491%	0.000%	
	0.459	-2.406	
positive	0	6	6
	5.789	159.211	
	0.000%	100.000%	3.509%
	0.000%	100.000%	
	0.000%	3.509%	
	-2.406	12.618	
Column Total	165	6	171
	96.491%	3.509%	

McNemar's Chi-squared test : Chi^2 = NaN d.f. = 1 p = NaN
McNemar's Chi-squared test : Chi^2 = NaN d.f. = 1 p = NaN
Minimum expected frequency: 0.2105263; Cells with Expected Frequency < 5: 1 of 4 (25%)
